# Determinants of enrolment in health insurance scheme among HIV patients attending a clinic in a tertiary hospital in South-eastern Nigeria

**DOI:** 10.4314/gmj.v57i1.3

**Published:** 2023-01

**Authors:** Chihurumnanya N Alo, Ifeyinwa C Akamike, Ijeoma N Okedo-Alex, Elizabeth U Nwonwu

**Affiliations:** 1 Department of Community Medicine, Alex Ekwueme Federal University Teaching Hospital Abakaliki Ebonyi State Nigeria; 2 Department of Community Medicine, Ebonyi State University, Abakaliki, Nigeria

**Keywords:** Health insurance, HIV, Enrolment, Nigeria

## Abstract

**Objective:**

The study aimed to assess the determinants of enrolment in health insurance schemes among people living with HIV.

**Design:**

The study was a cross-sectional study. A pre-tested interviewer-administered questionnaire was used to collect information from 371 HIV clients attending the clinic. Chi-square statistic was used for bi-variate analysis, and analytical decisions were considered significant at a p-value less than 0.05. Logistic regression was done to determine predictors of enrolment in health insurance.

**Setting:**

The study was carried out in the HIV clinic of Alex Ekwueme Federal University Teaching Hospital Abakaliki, Nigeria

**Participants:**

HIV clients attending a clinic

**Result:**

Mean age of respondents was 45.4±10.3, and 51.8% were males. Almost all the respondents were Christians. Only 47.7% were married, and most lived in the urban area. Over 70% had at least secondary education, and only 34.5% were civil servants. About 60% of the respondents were enrolled in a health insurance scheme. Being single (AOR: 0.374, CI:0.204-0.688), being self-employed (AOR: 4.088, CI: 2.315-7.217), having a smaller family size (AOR: 0.124, CI: 0.067-0.228), and having the higher income (AOR: 4.142, CI: 2.07-8.286) were predictors of enrolment in a health insurance scheme.

**Conclusion:**

The study has shown that enrolment in a health insurance scheme is high among PLHIV, and being single, self-employed, having a smaller family size, and having a higher monthly income are predictors of enrolment in the health insurance scheme. Increasing the number of dependants that can be enrolled so that larger families can be motivated to enrol in health insurance is recommended.

**Funding:**

None declared

## Introduction

In many low-income and middle-income countries (LMICs), raising sufficient funds for equitable health care has remained a challenge.^1^ Households in these countries lack adequate financial protection; most households depend on out-of-pocket payment for health services, resulting in financial catastrophe and impoverishing effects.^1^

Countries are encouraged to move towards universal health coverage to address the adverse effects of direct out-of-pocket payments.^1^,^2^ Social Health Insurance establishment is one of the ways through which universal health coverage can be achieved and is being advocated to ensure access to health services, especially for the underprivileged in less developed countries.^3^–^5^

Although there is increased effort to improve the provision of health care by many governments in developing countries, some of these countries still have difficulties achieving universal coverage.^2^,^6^ Nigeria is not an exception, as achieving a successful healthcare financing system has remained challenging.^7^ In Nigeria, high out-of-pocket spending continues to be a major challenge to health financing.^8^

Health insurance schemes usually cover only a small proportion of the population, particularly in low- and middle-income countries (LMICs), and people with high healthcare needs who require financial protection are least likely to be covered.^5^ HIV burden is high in these countries, and containing costs for treating people with HIV continues to pose a challenge.^9^

Improving access to health coverage for HIV clients through health insurance is one way of addressing this problem. Policies to expand insurance coverage to the uninsured HIV-positive population could save many lives.^10^ It has been documented that people who have HIV disease and who have access to health insurance usually have improved health outcomes, including viral suppression.^11^ HIV patients are among those with high healthcare requirements. People living with HIV(PLHIV) and ARVs may also need treatment for other illnesses, including HIV co-infections such as hepatitis C, drug-resistant tuberculosis, sexually transmitted diseases, cancer drugs including vaccines for human papillomavirus, and basic antibiotics for treatment of other infections.^9^ Consequently, people living with HIV need access to affordable medicine, especially with declining aid budgets and withdrawal of support by donor agencies.

Several studies have shown that despite high awareness of health insurance schemes among non-HIV clients, awareness does not translate to enrollment.^12^–^15^ Factors affecting enrolment in health insurance among non-HIV clients include low levels of income, lack of financial resources, poor healthcare quality, educational attainment, gender, age, and household size, and being a worker in the federal civil service.^1^,^14^,^16^ However, few studies have assessed the factors affecting enrolment in health insurance among PLHIV in Nigeria. This study aimed to assess the determinants of enrolment in health insurance among people living with HIV.

## Methods

### Survey setting and design

The study was conducted in Alex Ekwueme Federal University Teaching Hospital Abakaliki (AE-FUTHA), Ebonyi State, in the southeastern part of Nigeria. It is one of the tertiary hospitals in the state and is owned by the Federal Government. The hospital has over 4000 staff, comprising about 200 consultants in various specialties, and offers non-specialist and specialist health services, including HIV/AIDS and TB. AE-FUTHA is the largest comprehensive HIV/AIDs care centre in the state, and at the time of the study, the centre was supported by the Centre for Clinical Care and Research Nigeria (CCCRN).

About 1500 adult clients were enrolled and on treatment in the facility at the time of this study. The adult clinic runs weekly from Monday to Thursday with a daily average patient load of about 35. The study was a descriptive cross-sectional design with a population of adult (18 years and above) HIV patients attending the HIV clinic AE-FUTHA.

Those who were not yet on anti-retroviral therapy and were too ill to participate and those that did not give informed consent were excluded from the study.

### Study population and sampling technique

The sample size was calculated using a 95% confidence level, 5% precision and 40% as the proportion of clients enrolled in health insurance. The calculated sample size was 369; however, 371 clients participated. Systematic random sampling was used to select the participants at a sampling interval of 4 (k= 1500/371). Data was collected over three months (May-July 2018)

### Data collection

A pre-tested semi-structured questionnaire was used to collect information on the socio-demographic characteristics of respondents, their clinical characteristics, awareness of the health insurance scheme, and enrolment in a health insurance scheme. The research team included residents in the Department of Community Medicine, AE-FUTHA.

### Data management/analysis

The independent variables were socio-demographics (age, sex, marital status, residence, religion, family size, educational status, occupation, and monthly income), while the dependent variable was enrolment in a health insurance scheme. IBM Statistical Package for Social Sciences (SPSS) version 20 was used for the data entry and analysis. Frequencies and proportions were calculated for categorical variables, while means and standard deviations were calculated for numeric/quantitative variables. Descriptive statistics of the variables were presented using frequency tables. Chi-square was used for bi-variate analysis, and analytical decisions were considered significant at a p-value less than 0.05. Multivariable logistic regression for predictors of enrolment in health insurance was done using socio-demographic characteristics. The cut-off point for including variables in the regression model was p=0.1.

### Ethical considerations

Ethical clearance for this study was obtained from the Research and Ethics Committee of Alex Ekwueme Federal University Teaching Hospital Abakaliki (AE-FUTHA), Ebonyi State, Nigeria, with approval number: 24/01/2018-19/02/2018. Written informed consent was obtained, and confidentiality was ensured.

## Results

Table 1 shows that the mean age of respondents was 45.4±10.3, 51.8% were males, and almost all the respondents were Christians. Only 47.7% were married, and most lived in the urban area. Over 70% had at least secondary education, and only 34.5% were civil servants. More than half of the respondents (56.6%) had a family size of 5 or less, and only a few (17.5%) had a monthly income of greater than N40,000 ($111.1).

**Table 1:** Socio-demographic characteristics of respondents

Variable	Frequency(%) (n=371)
**Age (years) (Mean ±SD)**	45.4±10.3
**40 and less**	163(43.9)
**More than 40**	208(56.1)
**Gender**	
**Male**	192(51.8)
**Female**	179(48.2)
**Religion**	
**Christianity**	355(95.7)
**Islam**	16(4.3)
**Marital Status**	
**Never Married**	146(39.4)
**Married**	177(47.7)
**Divorced**	32(8.2)
**Widowed**	16(4.3)
**Residence**	
**Urban**	275(74.1)
**Rural**	96(25.9)
**Educational Status**	
**No formal education**	64(17.3)
**Primary school education**	32(8.6)
**Secondary school education**	96(25.9)
**Post-secondary school education**	179(48.2)
**Occupation**	
**Civil servant**	128(34.5)
**Artisan**	81(21.8)
**Trader**	82(22.1)
**Self-employed**	80(21.6)
**Family Size (Mean±SD)**	4.86±2.52
**5 or less**	210(56.6)
**6 or more**	161(43.4)
**Monthly Income (Naira) (Mean ±SD** **and range)**	N26,665.77±15,171.50 ($74.1±42.1) 2000–55,000 ($5.6- $152.8)
**Monthly income proportions**	
**<N20,000 ($55.6)**	145(39.1)
**N20,000-N40000 ($55.6-$111.1)**	161(43.4)
**>N40,000 ($111.1)**	65(17.5)

Table 2 shows that the number of years since diagnosis was 10 years and below for most respondents, and most had been on treatment for less than 10 years. Most respondents had no co-morbidity, and most rated their health as good.

**Table 2:** Clinical Characteristics of respondents

Variable	Frequency
**Number of years since diagnosis**	
**≤10 years**	273(73.6)
**>10 years**	98(26.4)
**Duration on treatment (years)**	
**≤5 years**	225(60.6)
**6–10 years**	81(21.8)
**≥10 years**	65(17.6)
**Co-morbidity**	
**No**	355(95.7)
**Yes**	16(4.3)
**Self-rated state of health**	
**Poor**	48(12.9)
**Good**	323(87.1)

Table 3 shows that the factors associated with enrolment include age, marital status, religion, employment status, family size, residence, state of health, co-morbidity, duration of treatment and number of years since diagnosis. Enrolment was higher among those less than 40 years, those never married, Christians, the self-employed, urban dwellers, those with good health, those without co-morbidities and those diagnosed less than 10 years ago.

**Table 3:** Factors associated with enrolment in health insurance

Variable	Enrolled (Percent)		Total (N = 371)	X^2^/Exact (*P*--value)
	
	No	Yes		
Gender				
Male	80(41.7)	112(58.3)	192	1.1(0.300)
Female	65(36.3)	114(63.7)	179	
Age (years)				
40 and less	49(30.1)	114(69.9)	163	9.9(0.002*)
More than 40	96(46.2)	112(53.8)	208	
Marital Status				
Never married and others	64(33.0)	130(67.0)	194	6.3(0.010*)
Married	81(45.8)	96(54.2)	177	
**Religion**				
Islam	16(100.0)	0(0)	16	FT(<0.001*)
Christianity	129(36.3)	226(63.7)	355	
**Employment** **status**				
Self-employed	81(33.3)	162(66.7)	243	9.7(0.003*)
Paid employment	64(50.0)	64(50.0)	128	
**Family size**				
5 or less	49(23.3)	161(76.7)	210	50.4(<0.001*)
>5	96(59.6)	65(40.4)	161	
**Highest educational level**				
Secondary and less	80(41.7)	112(58.3)	192	1.1(0.300)
Post-secondary/Tertiary	65(36.3)	114(63.7)	179	
				
Residence				
Urban	97(35.3)	178(64.7)	275	6.5(0.010*)
Rural	48(50.0)	48(50.0)	96	
Monthly income				
<N20,000 ($55.6)	64(44.1)	81(55.9)	145	2.6(0.100)
≥N20,000	85(35.8)	145(64.2)	226	
State of health				
Poor	48(100)	0(0)	48	FT(<0.001*)
Good	97(30)	226(70)	323	
Co-morbidity				
No	129(36.3)	226(63.7)	355	FT(<0.001*)
Yes	16(100)	0(0)	16	
Number of years since diagnosis				
≤10 years	96(35.2)	177(64.8)	273	6.7(0.010*)
>10years	49(50.0)	49(50.0)	98	
Duration on treatment				
≤5 years	48(21.3)	177(78.7)	225	79.6(<0.001*)
6–10 years	48(59.3)	33(40.7)	81	
>10years	49(75.4)	16(24.6)	65	

* Statistical significance FT: Fisher's exact test

Table 4 shows that the predictors of enrolment in insurance include marital status, employment status, family size and monthly income. Those that were married had 63% lower odds of being enrolled than those that were never married. Those that were self-employed were 4.088 times more likely to be enrolled than those with paid employment. Those with an income of N20,000 and above were four times more likely to be enrolled, while those with a family size of five and above had 87.6% lower odds of being enrolled.

**Table 4:** Logistic regression model for predictors of enrolment in health insurance

Variable	Adjusted Odds Ratio (CI)	Confidence Interval	P – value
**Age < 40years**	1.574	0.836–2.963	0.160
**40 years & more**			

**Marital status (Never married** **& Other/** **Married)**	0.374	0.204–0.688	<0.002*

**Employment status (Paid** **Employment/** **Self-employed)**	4.088	2.315–7.217	<0.001*
**Family Size(5 or less)**	0.124	0.067–0.228	<0.001*
**Family Size >5**			

**Monthly Income Status** **(<20,000)**	4.142	2.07–8.286	<0.001*
**(20,000** **and more)**			

**Residence (Urban/Rural)**	1.348	0.667–2.725	0.405

* Statistical significance

### Awareness and enrolment in Health Insurance

Eighty-three per cent of respondents were aware of the health insurance scheme, while more than half (61%) were enrolled in health insurance.

## Discussion

Our study assessed the level of enrolment in health insurance schemes and its associated determinants among people living with HIV in Alex Ekwueme Federal University Teaching Hospital, Abakaliki, Ebonyi State. The majority of the respondents were aware of the health insurance scheme. This finding can be explained by the fact that most respondents were urban dwellers who may have heard about health insurance from one source. In addition, this study was carried out in a tertiary health facility, and workers in a tertiary hospital who are enrolled in health insurance are likely to refer their relatives to receive treatment. This could have contributed to the high awareness. A high level of awareness has been reported in previous studies among PLHIV^15^ and non-HIV Clients.^12^–^14^ The study also revealed that most respondents were enrolled in a health insurance scheme.

Every employee in any federal establishment in Nigeria must be registered in the National Health Insurance Scheme. Each person enrolled is expected to cover four dependants.^17^ some of our respondents may be either spouses or dependants of those enrolled. This may be the reason for the high level of enrolment. This differs from what was reported in previous studies, which showed that awareness did not translate to enrollment.^13^,^14^ The present study may explain the disparity among HIV patients. HIV patients are more likely to be enrolled because they already bear the burden of ill health and must continue treatment throughout their lifetime. One way of reducing this burden is through enrolment in health insurance. The study further revealed that age, marital status, religion, employment status, family size, residence, state of health, co-morbidity, duration of treatment and number of years since diagnosis were significantly associated with enrolment in health insurance. Similarly, factors identified in previous studies among non-HIV clients include income, education, health risk, level of awareness, lack of financial resources, poor healthcare quality, gender, age, household size, and being a worker in the federal civil service.^1^,^14^,^16^,^18^

The predictors of enrolment found in this study include being single, being self-employed, having a smaller family size and having a higher monthly income. Those that were married had 63% lower odds of being enrolled than those that were single. This could be because single people may feel more secure being enrolled in insurance since they do not have a spouse to support them in the event of any illness.

Those that were self-employed were 4.088 times more likely to be enrolled than those with paid employment. This finding is quite surprising, considering the Nigerian health insurance scheme mainly covers those in the formal sector.^17^ This finding may be because the spouses of these self-employed respondents may already be enrolled in the health insurance scheme. Higher-income was also found to be a predictor of enrolment. This finding may be related to the fact that people with higher incomes are more likely to accept premium payments and still have enough from what is left. On the other hand, people with lower income may feel that they already have too little and will not permit any deductions. This finding agrees with the study in Ghana, which showed that wealthier individuals are more likely to enrol in the National Health Insurance Scheme.^19^ This also agrees with a study carried out in the USA among PLHIV, which found that people with higher income and longer duration of diagnosis were more likely to be enrolled.^15^ Those with less than five children were more likely to be enrolled, and this finding could be because of the limit to the number of children that can be enrolled in insurance schemes.^17^ The limitation of this current study is that it was carried out in only one health facility, so it may not be easy to generalise the findings.

## Conclusion

The study has shown that enrolment in a health insurance scheme is high among PLHIV and that being single, self-employed, having a smaller family size, and a higher monthly income are predictors of enrolment in the health insurance scheme. Policymakers may need to reduce the premium to accommodate people with lower incomes. It may also be necessary to increase the number of dependants that can be enrolled so that larger families can be motivated to enrol in health insurance
